# Nurse Practitioner Integration: Insights Into the Next Generation of Policy and Research

**DOI:** 10.34172/ijhpm.2023.7411

**Published:** 2023-06-06

**Authors:** Joshua Porat-Dahlerbruch, Lusine Poghosyan, Moriah Ellen

**Affiliations:** ^1^Department of Acute and Tertiary Care, School of Nursing, University of Pittsburgh, Pittsburgh, PA, USA; ^2^Department of Health Policy and Management, Guilford Glazer Faculty of Business & Faculty of Health Sciences, Ben-Gurion University of the Negev, Be’er Sheva, Israel; ^3^Israel Implementation Science and Policy Engagement Centre, Ben-Gurion University of the Negev, Be’er Sheva, Israel; ^4^Center for Healthcare Delivery Research and Innovations, School of Nursing, Columbia University, New York City, NY, USA; ^5^Department of Health Policy and Management, Mailman School of Public Health, Columbia University, New York City, NY, USA; ^6^Institute of Health Policy Management and Evaluation, Dalla Lana School of Public Health, University of Toronto, Toronto, ON, Canada

## Introduction

 Introduction of the nurse practitioner (NP) role is becoming an increasingly prevalent response around the world to address growing healthcare service and provider shortages (Box 1). Aside from positive patient outcomes associated with NPs,^2^ their holistic care is well-suited to meet population needs.^2^ Realizing these outcomes, nevertheless, cannot be achieved by introducing the NP role alone; it relies on successfully integrating NPs into all levels of the health system.

**Box 1. Definition Nurse Practitioners, Alternative Titles, and Countries With the Role**

**International Council of Nurses’ Definition of Nurse Practitioners**

“An advanced practice nurse who integrates clinical skills associated with nursing and medicine to assess, diagnose and manage patients in primary healthcare settings and acute care populations as well as ongoing care for populations with chronic illness”^1^

** Professional Titles Fitting with the International Council of Nurses’ Definition of NPs**

- Advanced practice nurse
- Nurse midwife
- NP
- Expert clinical nurse
- Advanced practice registered nurse

** Countries With an Established NP Role or in Preparation for the Introduction of an NP Role**

- Australia
- Cambodia
- Canada
- Czechia
- Finland
- Hungary
- India
- Ireland
- Israel
- Jamaica
- The Netherlands
- New Zealand
- Nigeria
- Norway
- South Africa
- Switzerland
- The United Kingdom
- The United States

-----------------

Abbreviation: NP, nurse practitioner.

*Note: *The box provides general information on the definition and breadth of the NP role around the world. This list is not inclusive of all professional titles meeting the International Council of Nurses definition of NPs,^1^ nor does it include all countries with an established NP role or in preparation for the introduction of the role.

NP integration can be defined as “a multi-level process of incorporating NPs into the health system to an extent at which they can function to the full scope of practice and contribute to patient, health system, and population needs.”^3^ Countries around the world vary significantly in their stage of NP integration — those at more advanced stages (eg, US and Canada) and those in earlier stages (eg, India and Czechia). While the term “integration” is often used interchangeably with “implementation,” the former insinuates complete incorporation into day-to-day function and has been defined in the literature.^3^

 Little research attention has been placed on identifying policies facilitating efficacious integration of NPs into health systems. Effective national and organizational policy-making relies on robust research identifying health system challenges affecting the integration of NPs.To guide investigation on NP integration, researchers require methodologies and frameworks/models for study design. As such, developing NP integration methods and frameworks/models may be critical for facilitating policy-making that better enables NPs to efficaciously meet growing demand for health services and improve care outcomes.

 In this viewpoint paper, we identify several key policy issues concerning the integration of NPs into health systems around the world and discuss recommendations for future research that could help address policy issues surrounding NP integration.

## Nurse Practitioner Integration Policy Issues

 NP integration involves incorporation of the NP role into three levels of the health system—*macro, meso, *and *micro*.^3^ The *macro* level refers to the national or jurisdictional level. *Meso* level denotes organizations, and *micro* level refers to interprofessional relationships and care teams. To highlight some existing policy issues that may benefit from research-informed knowledge, we drew on the international literature and organized key publications by health system level.

###  Macro

 In a review of policy levers for NP integration in OECD countries, Maier et al^2^ reported that medical workforce opposition, restrictive scope of practice laws, and inappropriate financing schemes hindered integration. A study in Israel reported that restructuring education programs will better prepare practice-ready NPs and advance integration.^4^ A review in India identified a poorly defined scope of practice for NPs, non-standardized education, poor clarity of professional pathways for NPs, and an absence of professional organizations for NPs.^5^

 These studies highlight a key concern regarding NP integration planning—a term which Bryant-Lukosius et al^6^ refer to as *ad hoc *integration. *Ad hoc *integration is described as introducing NPs into the workforce without sufficient attention to policies facilitating their integration, often resulting in limited autonomy in practice. The National Academies of Sciences, Engineering, and Medicine^7^ summarized that laws limiting autonomy are often rooted in non-evidence-based notions that NPs are less likely to provide high care quality. Scope of practice restrictions limit competition, lead to higher healthcare costs, and may impact care integration across settings.^7^ In the United States, state scope of practice laws have been in effect for decades and have proven difficult to retract—an exemplar of difficulties overcoming government policies once enacted and a “lesson learned” from a country with a longer history of NPs.

 To avoid the effects of *ad hoc* integration, national-level planning is critical for ensuring sustainability of the NP role. Key stakeholders, such as physician leaders, nursing organizations and policy-makers, should collaborate to develop a protected, clearly defined title and scope of practice. If lacking, physicians and employing organizations may be concerned about undue legal consequences, as reported in a scoping review of the NP integration literature in Atlantic Canada,^8^ or NPs may be incorporated into care teams as “physician extenders” instead of autonomous providers. NPs, in turn, may be deterred from seeking education and employment. Efficacious planning for NP integration, moreover, would likely benefit from a standardized, national accreditation system for education, clear communication of certification standards, and appropriate financing schemes to incentivize NP employment.^2^ These recommendations may be less feasible, however, for nations with fewer resources for centralized health governance or those in the early stages of NP integration. In such cases, efforts can be made to begin developing national infrastructure and pooling resources that will support gradual integration of NPs.

###  Meso

 Similar to governments at the *macro *level, organizations must plan and prepare for NP integration. A recent study in the United States found that NPs with poorer practice environments were four times less likely to report high care quality.^9^ These findings highlight a need to develop integration policies that improve the organizational climate. In Norway, a cross-sectional study revealed several organizational factors limiting the ability of NPs to practice to their intended scope —eg, insufficient infrastructure accommodating NP practice and lacking support from administrators.^10^ A scoping review on the integration of NPs into primary care settings found that organizational provision of mentorship and supervision is critical to successful NP integration.^11^ However, primary care organizations often lack such resources.^12^

###  Micro

 A cross-sectional survey of NPs and physicians in primary care practices in New York found that NP-physician teamwork affects clinician job satisfaction and intent to leave.^13^ Ethnographic research in New Zealand found that NP practice is restricted by administrative and physician colleagues who often consider NPs to be a physician “substitute.”^14^ These studies underscore the importance of interprofessional relationships for facilitating NP integration. A recent scoping review on integration into primary care settings found that physicians lacked confidence regarding the adequacy of NP training/education and readiness to acquire provider authorities.^11^ These concerns may be rooted in poor knowledge of the NP role and/or training and licensing requirements.

###  Nations in Early Stages of Role Development

 Many of the conclusions from the literature are less relevant for nations in the early stages of NP integration. Several nations lack centralized workforce planning, primary care infrastructure, nursing accreditation bodies, and educational institutions.^1,2^ As such, *ad hoc* integration and other barriers are unavoidable. Developing the necessary infrastructure takes time. Furthermore, the literature on NP integration in these early-stage settings is scarce, rendering a lack of policy guidance. In a recent review of the international NP integration literature including 77 publications, only 23% were from outside the United States and Canada.^3^ The dearth may be due to lacking published methodologies and frameworks that can be adopted by local researchers in countries in the early stages of integration.

###  Key Takeaways

 These studies identify just a few issues hindering the integration of NPs. As a result of prevalent *ad hoc* integration and insufficient *macro*-level planning, NPs are likely not maximally contributing to improved patient outcomes and reduced provider shortages. Without robust, *macro-*level legislative/regulatory support, efficacious NP integration cannot occur at *meso* nor *micro *levels. In other words, policies trickle down. To create institutional policies and appropriate infrastructure facilitating the incorporation of NPs into care models, care organizations and health professional colleagues rely on clear guidance from the *macro* level on the scope of NP practice. The current literature, furthermore, tends to focus on nations with more healthcare resources and lacks syntheses of the full spectrum of policy issues affecting NP integration at both earlier and advanced stages. This is a dearth in the literature and the science.

## A Call for Research on Nurse Practitioner Integration

 Inadequate NP integration planning and policy development can be attributed to a lack of comprehensive research on the topic.^2,5,11^ Here, we discuss past and current trends in NP integration research and recommendations moving forward.

###  Current and Past Research Direction

 We posit that the lack of synthesized research and theoretical development on the topic of NP integration roots largely in the current methodological focus of NP health services research. The field tends to focus on the contributions of NPs to care outcomes based on specific settings^15^ or organizational characteristics^16^ — eg, work environment. It is indeed critical to understand whether NP care is linked to improved outcomes since care quality evidence can prove beneficial for convincing stakeholders to adopt the role. However, outcomes research is mainly intended to measure the effects of the presence of NPs. It may be appropriate where NPs are integrated efficaciously and are able to practice to their full, intended scope — eg, many settings in North America. Outcomes research may be premature, however, where NPs are not yet fully integrated. In such cases, NP care does not likely contribute to outcomes at a measurable magnitude. Research focus should be expanded to develop the health services research discipline and inform policy in settings where NP integration is less advanced.

 While existing NP integration models/frameworks and methodologies may prove informative, they are not comprehensive and require further development for operationalization. The Patient-Centered Process for Advanced Practice Development^17^ is a widely cited, broad framework including nine steps guiding the integration of NPs with a focus on population needs and stakeholder engagement. However, the science would benefit from a framework/model covering all three health system levels. The NP Integration Model^3^ addressed the three health system levels from an international lens. The model, however, is at a conceptual stage and requires empirical testing for applicable use in policy and research. Finally, Sangster-Gormley et al^18^ reported several mixed methods approaches for evaluating NP integration progress at a jurisdictional level. These evaluation methods would complement a model also describing the process of integration.

###  Future Research Direction

 In a review of *macro*-level policies affecting the integration of NPs, Maier et al^2^ concluded that NP integration is a process with similar attributes throughout the world. We posit, accordingly, that it is possible to develop a comprehensive, global model for NP integration. Policy-makers and organizational administrators can refine this global model for application in their specific context — ie, a nation, jurisdiction, or organization.

 An NP integration model would need to delineate the indications for introducing NPs, the attributes of the process, facilitators/barriers to integration, and expected outcomes of NP integration. A conceptual model can be developed through international literature reviews and conceptual refinement through international expert consensus. For operational use, methods for identifying integration facilitators/barriers and evaluating integration would need to be developed—eg, a survey intended for one population and later validated among other populations. Finally, practical policy solutions mapped to facilitators/barriers would aid in policy-making and can be developed through international qualitative research with policy-makers.

 NP health services researchers would need to derive novel research methodologies to produce a comprehensive NP integration model. Implementation science, which focuses on the translation of evidence-based interventions into practice, may prove informative. We posit that NPs, too, are an evidence-based intervention that, once integrated efficaciously, can improve several health system outcomes — eg, reduced health disparities.^19^ Still, to achieve these outcomes, policies must facilitate the effective integration of NPs into all levels of the health system.

 Implementation science would be particularly informative for developing an NP integration model since its frameworks/models are broad and tailorable to allow adaptability by multiple countries and contexts.^20^ An implementation science-based NP integration framework, for instance, could be applied in a decentralized health system where comprehensive health services research is notoriously challenging due to differing legal and regulatory policies.^2^ Here, researchers could exclude the facilitators/barriers irrelevant for a specific jurisdiction under study. Policy-makers planning for integration could tailor the process to meet the population care needs in their region. Several existing implementation science frameworks/models provide guidelines for application across settings.^20^

## Conclusion

 Although the number of NPs is growing internationally, little attention has been given to policies improving their integration into health systems. Without research to aid policy-makers and administrators in understanding NP integration, policies will likely not facilitate NP contribution to improved health outcomes. To maximize the efficacy of the healthcare provider workforce and address care needs, novel research is needed to develop a model to guide NP integration. Future publications should delineate steps for developing an NP integration model.

## Ethical issues

 Not applicable.

## Competing interests

 Authors declare that they have no competing interests.

## Funding

 This work was supported by the US-Israel Fulbright Commission [Grant Number 11541-IS].
